# B Cell Signaling and Activation in Autoimmunity

**DOI:** 10.3390/cells12030499

**Published:** 2023-02-03

**Authors:** Rudi W. Hendriks, Odilia B. J. Corneth

**Affiliations:** Department of Pulmonary Medicine, Erasmus MC, University Medical Center Rotterdam, 3000 CA Rotterdam, The Netherlands

Autoreactive B cells play a key role in the initiation or aggravation of many systemic and tissue-specific autoimmune disorders. This is not only due to their inadvertent differentiation into auto-antibody-producing cells, but also because they produce pro-inflammatory cytokines and act as antigen-presenting cells. As a result, B cells can engage T cells in an auto-immune response. Although the molecular mechanisms that underlie the pathogenic dysregulation of B cell activity remain poorly characterized, numerous lines of evidence support the unbalanced activation of various signaling pathways in B cells as a critical driver of autoimmune disease.

In this Special Issue of *Cells*, both reviews and original articles address how aberrant signaling events in various pathways in B cells contribute to autoimmune pathology. The majority of B cells that develop in the bone marrow show some level of autoreactivity. In healthy individuals, many autoreactive B cells are removed from the repertoire by central tolerance mechanisms. As detailed in Bonasia et al. [1] and Corneth et al. [2], these mechanisms include clonal deletion, receptor editing and anergy, all of which are guided by the strength of signals downstream from the B cell antigen receptor (BCR) recognizing self-antigens in the bone marrow. Nevertheless, a substantial fraction of autoreactive B cells still leave the bone marrow. Activation and differentiation of these autoreactive B cells following a breach in tolerance checkpoints in germinal centers are common mechanisms across many autoimmune diseases. The reviews give an overview of the signaling pathways in B cells that are thought to be responsible for the defective selection and pathogenic activation of B cells, thereby fueling T–B cell interaction in germinal centers. Moreover, Bonasia et al. [1] indicate possible therapeutic targets to eliminate or prevent the activation of autoreactive B cells, not only by interfering with the function of particular signaling molecules in B cells (which will essentially not be specific for autoreactive B cells), but also by genetically engineered T cells aiming to specifically eliminate pathogenic self-reactive B cells.

Genome-wide association studies (GWAS) have provided genetic evidence for the involvement of a range of genes associated with particular autoimmune diseases. Many of the susceptibility genes uncovered by GWAS encode signaling proteins. It remains challenging, however, to unravel the molecular mechanisms involved in disease pathology, mainly because most genetic variants are located in non-coding regions. One of the exceptions may be the B cell scaffold protein with ankyrin repeats (*BANK1*) gene for which an association was found with systemic lupus erythematosus (SLE) as well as other autoimmune diseases. In this Special Issue, Gomez Hernandez et al. [3] review the function of BANK1 in c and Toll-like receptor signaling. In particular, they discuss the functional consequences of a rare coding variant, W40C, as well as risk alleles, in addition to the knowledge derived from animal models. Nevertheless, as is the case for many other signaling molecules, functional studies are complicated by the expression of BANK1 in multiple cell types of the immune system and the involvement of BANK1 in multiple signaling pathways. Regarding the development of therapeutic strategies for autoimmune disease, these characteristics of signaling molecules may hamper the clinical application of even highly specific small molecule inhibitors. On the other hand, the concomitant blocking of BCR-driven activation of B cells and FcγRIII-induced production of pro-inflammatory cytokines in macrophages by a Bruton’s tyrosine kinase (BTK) inhibitor provided a compelling rationale for targeting BTK in rheumatoid arthritis [4].

Targeting B cell signaling by various inhibitors is also discussed by Fetter et al. [5], particularly in the context of a range of autoimmune conditions with cutaneous manifestations. An important aspect of this disease group is that next to antibody production, the auto-reactive skin-associated B cells in chronically inflamed skin are a driving force in the formation and maintenance of tertiary lymphoid structures because of their ability to present antigens to T cells and to create a pro-inflammatory microenvironment. This warrants the investigation of B cells even in cutaneous autoimmune diseases lacking auto-antibody formation. In parallel, alterations in B cell signaling pathways also play an important role in conditions not typically regarded as autoimmune diseases. In this Special Issue, Harder et al. [6] demonstrate that phosphoinositide-3-kinase (PI3K) signaling is severely dysregulated in the B cells of a subgroup of patients with common variable immunodeficiency (CVID), a primary antibody deficiency whereby a substantial fraction of patients display symptoms of autoimmunity. Increased basal and disturbed BCR-activated PI3K signaling was found, especially in the T-bet^high^CD21^low^ age-associated B cell subset, which is known to accumulate in peripheral blood in CVID patients as well as in patients with autoimmune diseases such as SLE and rheumatoid arthritis (RA). Furthermore, we observed enhanced BCR signaling in idiopathic pulmonary fibrosis (IPF), in which there is some evidence for a role of B cells and autoimmunity [7]. Anti-Ig-induced phosphorylation of BTK and phospholipase Cγ2 (PLCγ2) was increased in naive but not in memory B cells in the circulation of patients with IPF. Interestingly, the treatment of IPF patients with the anti-fibrotic tyrosine kinase inhibitor nintedanib induced major changes in BCR signaling, whereby patients who showed high BCR-driven phosphorylation of spleen tyrosine kinase (SYK), PLCγ2 and PI3K before treatment showed low phosphorylation after treatment, and vice versa. Previously, we also found that treatment with BTK inhibitors in mouse models resulted in rewiring of proximal and distal BCR signaling in healthy and malignant cells [8]. Therefore, detailed analyses of B cell signaling in clinical trials of small molecule inhibitors targeting signaling pathways in autoimmune disease will be of great added value. In this context, it is important that sensitive single-cell-based intracellular flow cytometric methods to quantify the phosphorylation of signaling molecules or the expression levels of (downstream) transcription factors continue to be developed and improved, as presented in this issue by Marsman et al. [9]. Moreover, the field will particularly benefit from novel technology that facilitates the characterization and purification of auto-antigen-specific B cells that are typically present at very low frequencies [10,11]. Such in-depth analyses will lead to the identification of specific therapeutic targets and may guide the selection of specific small molecule inhibitors for evaluation in clinical trials in various autoimmune diseases.
